# Abundance of female-biased and paucity of male-biased somatically expressed genes on the mouse X-chromosome

**DOI:** 10.1186/1471-2164-13-607

**Published:** 2012-11-10

**Authors:** Björn Reinius, Martin M Johansson, Katarzyna J Radomska, Edward H Morrow, Gaurav K Pandey, Chandrasekhar Kanduri, Rickard Sandberg, Robert W Williams, Elena Jazin

**Affiliations:** 1 Department of Organismal Biology, Evolutionary Biology Centre, Uppsala University, Uppsala, Sweden; 2 Ludwig Institute for Cancer Research, Stockholm, Sweden; 3 Department of Cell and Molecular Biology, Karolinska Institutet, Stockholm, Sweden; 4 School of Life Sciences, University of Sussex, Brighton, United Kingdom; 5 Department of Genetics and Pathology, Rudbeck Laboratory, Uppsala University, Uppsala, Sweden; 6 Department of Medical and Clinical Genetics, Department of Biomedicine, The Sahlgrenska Academy, Gothenburg University, Gothenburg, Sweden; 7 Department of Anatomy and Neurobiology, University of Tennessee, Knoxville, USA

**Keywords:** X-chromosome, Sex chromosome, Somatic, Gene expression, Sexual antagonism, Sexual selection, Gender, Sex-bias, Female-bias, Male-bias, Sexual dimorphism, Dosage compensation, X-inactivation, Escape, Feminisation, Masculinisation, De-masculinisation, Microarray, Non-coding RNA, lncRNA, *Tmem29*, *Kdm5c*, *Xist*

## Abstract

**Background:**

Empirical evaluations of sexually dimorphic expression of genes on the mammalian X-chromosome are needed to understand the evolutionary forces and the gene-regulatory mechanisms controlling this chromosome. We performed a large-scale sex-bias expression analysis of genes on the X-chromosome in six different somatic tissues from mouse.

**Results:**

Our results show that the mouse X-chromosome is enriched with female-biased genes and depleted of male-biased genes. This suggests that feminisation as well as de-masculinisation of the X-chromosome has occurred in terms of gene expression in non-reproductive tissues. Several mechanisms may be responsible for the control of female-biased expression on chromosome X, and escape from X-inactivation is a main candidate. We confirmed escape in case of *Tmem29* using RNA-FISH analysis. In addition, we identified novel female-biased non-coding transcripts located in the same female-biased cluster as the well-known coding X-inactivation escapee *Kdm5c*, likely transcribed from the transition-region between active and silenced domains. We also found that previously known escapees only partially explained the overrepresentation of female-biased X-genes, particularly for tissue-specific female-biased genes. Therefore, the gene set we have identified contains tissue-specific escapees and/or genes controlled by other sexually skewed regulatory mechanisms. Analysis of gene age showed that evolutionarily old X-genes (>100 myr, preceding the radiation of placental mammals) are more frequently female-biased than younger genes.

**Conclusion:**

Altogether, our results have implications for understanding both gene regulation and gene evolution of mammalian X-chromosomes, and suggest that the final result in terms of the X-gene composition (masculinisation versus feminisation) is a compromise between different evolutionary forces acting on reproductive and somatic tissues.

## Background

Females and males have different routes to maximize fitness and therefore the two sexes have different optimal transcriptomes. The X-chromosome is of particular interest in terms of understanding the evolution of sexual dimorphism [1]. Heterogamety in males (XY) and homogamety in females (XX) makes the X-chromosome a unique environment that can expose sexual genomic conflict. Sex-specific selection pressures can favour phenotypic divergence between the sexes [2]. When an allele has opposite effects on fitness in males and females (intralocus conflict) divergent evolution of associated traits is likely to be constrained, and sexual antagonism in allele selection can persist over long timescales [3-6]. Unlike autosomes, the mammalian X-chromosomes have spent approximately 2/3 of their evolutionary time in females, as compared to 1/3 in males. Consequently, it might be predicted that the X-chromosome should be enriched with female-beneficial rather than male-beneficial alleles [7,8], although this is also dependent on the dominance state of the alleles. Rice has argued that heterogamety puts X under direct selection in males, and alleles that confer differential fitness, particularly as recessives, will be strongly selected in males, leading to the gradual masculinisation of X during evolution [9].

Sequencing of the mammalian X-chromosomes and genome-wide expression profiling are now providing empirical data, shedding new light on these ideas. Indeed, sex-biased expression is at present commonly used as an indicator of previously resolved sexual antagonism at the genomic level [1]. For example, gene expression studies have revealed feminisation of the X in *Drosophila melanogaster*[10] and *Caenohabditis elegans*[11]. However, results from studies of mammalian X-chromosomes have been partly conflicting. For example, the mouse X-chromosome has been suggested to be both feminised and masculinised for genes expressed in tissues related to reproduction [8]. Extensive sex-biased expression also occurs in non-reproductive tissues [12]. Therefore comprehensive analyses of sexually dimorphic gene expression in somatic tissues could provide further insight into the sex-biased properties of the mammalian X-chromosome.

The current model of the evolution of the XY system describes a chain of events in which the X and Y evolved from a pair of autosomes [7]. In brief, this process involves the establishment of a testis determining factor (TDF) on the proto Y-chromosome, the repression of X/Y recombination to conserve linkage between the TDF and male-beneficial genes on Y, and the consequential erosion of Y which cause the need for up-regulation of X-linked genes in males to restore the X-chromosome:Autosomal ratio of critical genes [13,14]. Mammalian females have a double dose of all genes located on the non-recombining region of the X relative to males. To reach an equal expression level of X-genes between the sexes, mechanisms evolved to transcriptionally silence one of the two copies of genes encoded on the Xs in females. This is achieved by a chromosome-wide mechanism termed X-chromosome inactivation (XCI) [15]. In female cells, one X is transcriptionally inactive (Xi) while the other remains active (Xa). The selection of Xi and Xa occurs at random in each cell during early development and the silenced state of one X is maintained during cell division throughout adulthood. XCI makes general theoretical predictions of masculinisation versus feminisation of the gene content on X complex [16]. For example, for circulating gene products females might be effectively heterozygous while for intracellular gene products they might be effectively hemizygous chimeras. Empirical investigations of sex-biased expression of the X-chromosome are therefore essential for understanding the evolution of this chromosome. Furthermore, the silence of Xi is not complete. It has been known since the 1970s that some genes "escape" XCI and are expressed from both X-alleles [17,18]. Early investigations showed that “escapees” are located in disjoint parts of the X-chromosome, and that escape proceeds by a mechanism that allows for considerable autonomy between different genes and regions on the X-chromosome [19,20]. Now, forty years later, we know many more genes that escape XCI in humans and mouse, but the molecular mechanisms determining escape remain unclear. It is established that escape from XCI can produce gene expression differences between males and females, and that this is one important mechanism for explaining how female and male phenotypes can be obtained from two similar genomes [1,21]. Escape from XCI is not the only mechanism that can produce sex-biased expression on X. A careful and comprehensive combined analysis of gene content, escape of inactivation, imprinting as well as other allelic asymmetries and their epigenetic control mechanisms will be required in order to understand the selective forces operating on X. For this, robust identification of sex-biased genes located on the X-chromosome is needed. Several previous studies concerned the distribution and expression of X-genes with functions in reproductive tissues, but equally extensive studies of sex-biased expression in somatic tissues are still lacking. To remedy this, we performed large-scale analyses of microarray hybridisations with male and female RNA from multiple somatic mouse tissues including kidney, liver, lung, eye, and two brain regions: the striatum and hippocampus.

## Results

### High-resolution screening for sex-biased X-genes in somatic tissues

**Figure 1:**
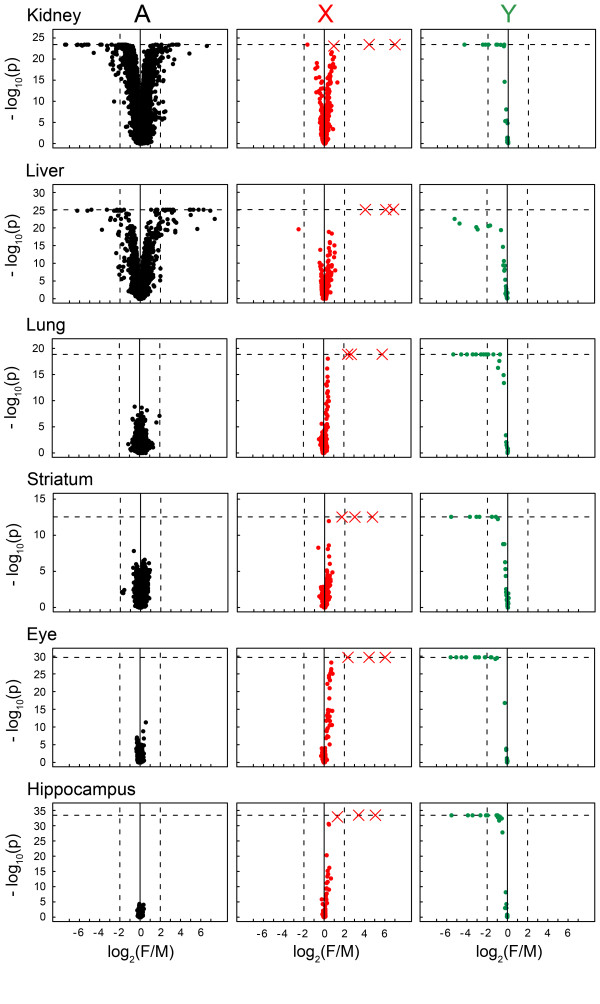
**Overview of the microarray results.** The data is separated according to chromosomal mapping: Autosomes (A), X-chromosome (X) and Y-chromosome (Y). On the x-axis: mean female/male log_2_ fold-changes; negative values represent male-bias and positive values represent female-bias. On the y-axis: -log_10_ p-values (two-sided Wilcoxon test). Each dot represents a probe and the data points marked with crosses represent probes for *Xist*. The horizontal dashed lines indicate the significance level at which probe intensities presented a non-overlapping distribution between the sexes; probes lying on this line show higher expression in one sex relative to the other in all possible combinations of individual comparisons. The vertical dashed lines mark the fold-change +/−4 (i.e. 2 on log_2_ scale), included in the figure for the sake of comparisons.

Our first goal was to build a comprehensive list of genes encoded on the mouse X-chromosome with sex-biased expression in somatic tissues. To provide sufficient statistical power to detect even small expression differences, we analyzed a large collection of oligonucleotide microarrays (n_arrays_=728, Affymetrix Mouse 430v.2) hybridized with RNA samples obtained from six somatic tissues: kidney (n_arrays_=152, with n_female arrays_: n_male arrays_ equal to 99:53), liver (88, 44:44), lung (67, 33:34), striatum (46, 25:21), eye (176, 88:88) and hippocampus (199, 101:98). To identify differentially expressed genes, we applied Wilcoxon rank-sum test (results from parametric tests are broadly similar, see Methods). The result from this analysis is graphically presented in Figure 1, including data for X, Y and autosomal chromosomes. Table 1 gives an overview of the results obtained at the significance threshold p<0.001. Detailed data for all individual genes and tissues are found in Additional file 1 and Additional file 2.

**Table 1:** Summary of the microarray results

**Tissue**	**Probes**	**All probes**			**Autosomes**			**X-chromosome**			**Y-chromosome**			**Location N/A**		
		**Tot**	**F**	**M**	**Tot**	**F**	**M**	**Tot**	**F**	**M**	**Tot**	**F**	**M**	**Tot**	**F**	**M**
Kidney	#	8153	4360	3793	7825	4157	3668	287	196	91	15	0	15	26	7	19
	%	18.1	9.67	8.41	18.2	9.67	8.54	20.1	13.7	6.38	45.5	0	45.5	3.87	1.04	2.83
Liver	#	5193	2420	2773	4961	2296	2665	176	111	65	15	0	15	41	13	28
	%	11.5	5.37	6.15	11.5	5.34	6.20	12.3	7.78	4.56	45.5	0	45.5	6.11	1.94	4.17
Lung	#	563	304	259	491	259	232	48	42	6	16	0	16	8	3	5
	%	1.25	0.67	0.57	1.14	0.60	0.54	3.36	2.94	0.42	48.5	0	48.5	1.19	0.45	0.75
Striatum	#	747	541	206	690	497	193	38	37	1	12	0	12	7	7	0
	%	1.66	1.20	0.46	1.61	1.16	0.45	2.66	2.59	0.07	36.4	0	36.4	1.04	1.04	0
Eye	#	106	65	41	49	28	21	38	36	2	17	0	17	2	1	1
	%	0.24	0.14	0.09	0.11	0.07	0.05	2.66	2.52	0.14	51.5	0	51.5	0.30	0.15	0.15
Hippocampus	#	50	26	24	8	2	6	25	23	2	16	0	16	1	1	0
	%	0.11	0.06	0.05	0.02	0.00	0.01	1.75	1.61	0.14	48.5	0	48.5	0.15	0.15	0

The table presents the number (#) and the percentage (%) of significantly sex-biased assayed probes (p<0.001, two-sided Wilcoxon test) separated by chromosomal location. Abbreviations: *Tot* Total, *F* female-biased, *M* male-biased, *N/A* chromosomal location not available. Complete data for all individual probes is available in Additional file 1 and Additional file 2.

A first observation was that the overall percentage of sex-biased transcripts encoded on the X-chromosome was higher than the percentage of sex-biased transcripts encoded on the autosomes, and this observation was consistent in all tissues (See columns labelled “Autosomes Tot” and “X-chromosome Tot”, Table 1). Therefore, it can be concluded that the X-chromosome is a chromosomal location overrepresented for sex-biased genes, in agreement with previous results [12]. Also in line with previous investigations, we observed substantial tissue differences in the number of sex-biased genes (Table 1). This was the case when considering only the X-chromosome (ranging from 25 to 287 transcripts) as well as when considering autosomes (8–7825 transcripts), while the number of Y-linked genes remained nearly constant between the tissues analysed (12–17 transcripts).

### Large number of genes with small magnitude sex-biased expression on X

**Figure 2:**
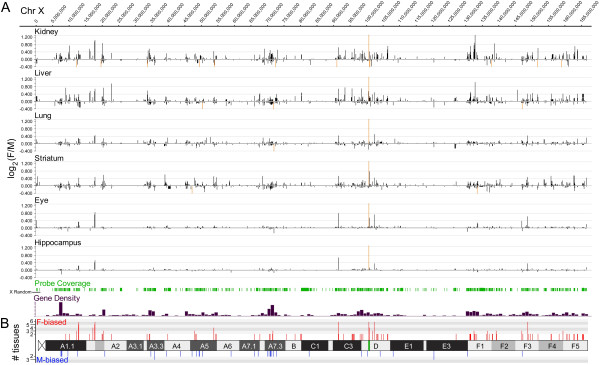
**Localisation of sex-biased genes on the mouse X-chromosome.A**: The female/male relative expression levels of all assayed transcripts on the mouse X-chromosome is shown for the six tissues included in the study. The heights of the bars indicate mean log_2_ female/male fold-change and the x-axis corresponds to spatial location on X (NCBI37/mm9 genome assembly). Orange bars indicate a fold-change beyond the given y-axis limit. Probe coverage (Affymetrix M430v2) and gene density (UCSC Browser, NCBI37/mm9, 10 Mb bins) is shown below. **B**: The distribution of transcripts with consistent female-bias (red) and male-bias (blue) in more than two tissues is shown. The heights of the bars indicate the number of tissues wherein the transcripts were sex-biased. The corresponding probes are listed in Table 2. The green belt on the D-band marks the X-inactivation center.

We found that while sex-biased genes located on X are numerous, the magnitude of their sex differences was small, and this was valid for all somatic tissues investigated. For example, while 80 autosomal transcripts showed sex differences larger than 4-fold in the kidney, not a single transcript with such a large difference was found on the X-chromosome when *Xist* (X-inactive specific transcript, “Master regulator” of XCI) was excluded (Additional file 1). In the liver, 53 autosomal transcripts were sex-biased beyond 4-fold, while only a single male-biased X-linked gene (*Alas2*) showed differences beyond 4-fold (*Xist* excluded). These small differences are better illustrated in Figure 1, where only three crosses, all of them representing probes for parts of the *Xist* gene, resulted in female-bias greater than 4-fold. At the same time, the figure shows results for many autosomal transcripts with large sexual expression differences, particularly in the liver and kidney. Genes encoded by the Y-chromosome also resulted in large differences in all tissues as expected, since they are only expressed in males (Figure 1). The spatial distribution of sex-biased genes along the X-chromosome is illustrated in Figure 2A, where it can be observed that their distribution is widespread over the chromosome rather than concentrated on specific chromosomal segments, and does not significantly diverge from the overall gene density on the X.

### Abundance of female-biased and paucity of male-biased genes on X

**Figure 3:**
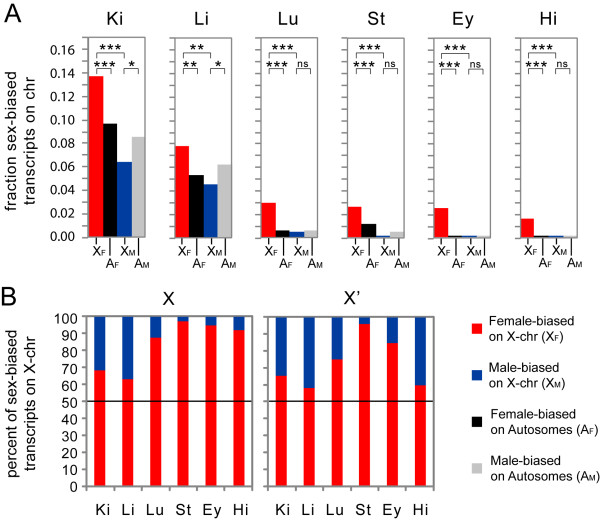
**Abundance of female-biased and paucity of male-biased genes on X.A**: Comparisons of the fraction of female-biased and male-biased transcripts within the X-chromosome and between the X-chromosome and autosomes in six somatic tissues: kidney (Ki), liver (Li), lung (Lu), striatum (St), eye (Ey) and hippocampus (Hi). P-values for differences in frequencies; ns: not significant, *: p<0.01, **: p<0.001, ***: p<0.0001 (two-sided Fisher’s exact test). **B**: The relative overrepresentation of female-biased as compared to male-biased transcripts on the mouse X-chromosome is illustrated (**X**). Overrepresentation of female-biased transcripts on X mostly remained after exclusion of all known XCI escapee genes (**X**’). The data presented in **A** and **B** corresponds to probes selected at p<0.001 (two-sided Wilcoxon test).

To study the relative allocation of male- and female-biased genes, we compared their frequencies on the X-chromosome. We found significantly more transcripts with female-bias than with male-bias in all six somatic tissues examined (Figure 3A, comparison X_F_*vs*. X_M_, p<0.001). Next, we compared the X with autosomes, and found female-biased genes to be more abundant on the X in all tissues (Figure 3A, comparison X_F_*vs*. A_F_, p<0.001). More remarkable, in the most sexually dimorphic somatic tissues analysed: the kidney and liver, we found a significant depletion of male-biased genes on the X relative to the frequencies on autosomes (Figure 3A, comparison X_M_*vs*. A_M_, kidney: 25% relative depletion, p= 0.0032 liver: 27% relative depletion, p= 0.0099). These observations provide the first evidence of de-masculinisation of a mammalian X-chromosome in terms of X-gene expression in somatic tissues. The overall frequencies of male-biased genes in the remaining four tissues were too low to allow similar meaningful comparisons between X and autosomes in those tissues.

To evaluate whether biallelic expression of genes previously known to escape XCI could explain the excess of female-biased transcripts observed on X, we compared the frequencies of male- and female-biased X-genes (Figure 3B, left panel) with the frequencies of sex-biased genes excluding previously known mouse escapees (Figure 3B, right panel). As expected, the exclusion of escapees reduced the female to male imbalance on the X, however, a substantial excess of female-biased genes remained. This suggests the existence of novel tissue-specific escapees in our gene sets, and/or other sex-skewed mechanisms exist that regulate the expression of the remaining female-biased genes.

### Small female-biased gene clusters on X

We have previously identified small female-biased gene clusters on the mouse X-chromosome, including female-biased long non-coding RNAs (lncRNAs) and protein coding genes that escape XCI, raising the possibility that such lncRNAs might be involved in the epigenetic regulation of escape domains [22]. Using the more extensive datasets included in the current study, we searched for additional female-biased clusters or additional genes in previously found clusters. To do this, we performed a second analysis in which the significance cut-off was relaxed from p<0.001 to p<0.01, increasing the sensitivity to detect sex-biased genes. At the same time, we only selected genes with consistent bias in more than one tissue, decreasing the expected number of false positives. The result from this analysis is shown in Table 2, containing 94 genes (and 8 additional non-annotated intronic sequences) with female-bias, and only 33 genes (and 2 intronic sequences) with male-bias. The spatial distribution of these genes on the X-chromosome is shown in Figure 2B. Female-biased genes located in close proximity to each other (defined as less than 50 kb) are marked “*af*” on the right side of Table 2. The table then shows 6 clusters, and each of them, with the exception of cluster “*e*”, containing an lncRNA. One of the female-biased clusters “*f*” deserves special attention because it includes several novel non-coding genes. In addition to the known escapees *Kdm5c* (coding) and *D930009K15Rik* (non-coding), three additional female-biased 3’-located non-coding genes were herein identified in this cluster, including *2810454L23Rik*, *1441020*_*at* (intergenic) and *2900056M20Rik*.

**Table 2:** Genes with sex-bias in multiple tissues

**A. Female-biased genes**											
**ProbeSet ID**	**Start align**	**Stop align**	**Ki**	**Li**	**Lu**	**St**	**Ey**	**Hi**	**Gene symbol**		
1426269_at	Rnd 10740	Rnd 39163	ns	*1.15*	**1.16**	ns	**1.31**	ns	*Vamp7*	1	
1452007_at	Rnd 10740	Rnd 39163	ns	*1.21*	ns	ns	**1.32**	ns	*Vamp7*	1	
1436778_at	9012379	9015421	ns	**1.65**	*1.19*	ns	ns	ns	*Cybb*	2	
1436779_at	9012379	9015421	ns	**1.25**	**1.16**	ns	ns	ns	*Cybb*	2	
1448737_at	10062241	10173728	**1.66**	**1.29**	ns	ns	ns	ns	*Tspan7*	3	
1433867_at	12232009	12232793	**1.08**	*1.14*	ns	ns	*1.05*	ns	1810030O07Rik*	4	
1416467_at	12858147	12870179	**1.21**	*1.18*	**1.22**	ns	**1.26**	**1.16**	Ddx3x*	5	
1423042_at	12868948	12871177	**1.35**	**1.21**	**1.19**	**1.35**	**1.24**	**1.18**	Ddx3x*	5	
1423043_s_at	12868948	12871177	**1.37**	**1.32**	**1.14**	ns	**1.21**	**1.18**	Ddx3x*	5	
1428667_at	16196827	16262870	**1.47**	**1.36**	ns	ns	ns	ns	*Maoa*	6	
1442676_at	16262773	16263384	**1.05**	**1.14**	ns	ns	ns	ns	*Maoa*	6	
1424215_at	17133689	17149423	**1.14**	**1.17**	*1.10*	ns	**1.06**	ns	*Fundc1*	7	
1427672_a_at	17740544	17855992	**1.94**	**1.45**	**1.32**	ns	**1.64**	**1.32**	Kdm6a*	8	
1446278_at	17804913	17805592	**1.18**	**1.18**	**1.36**	ns	**1.46**	**1.15**	*--- (Kdm6a intron)*	8i	
1446234_at	17805383	17806066	**1.53**	**1.32**	**1.33**	ns	**1.76**	**1.38**	*--- (Kdm6a intron)*	8i	
1427235_at	17818825	17856644	**2.05**	**1.60**	**1.31**	**1.38**	**1.80**	**1.43**	Kdm6a*	8	
1445198_at	17854669	17855386	**1.09**	**1.25**	**1.26**	ns	**1.49**	**1.13**	Kdm6a*	8	
1434061_at	19976041	19977980	*1.19*	*1.19*	ns	ns	ns	ns	*Rp2h*	9	
1454816_at	19976041	19977980	**1.11**	*1.10*	ns	ns	ns	ns	*Rp2h*	9	
1448852_at	20126913	20139212	**1.82**	**1.20**	ns	ns	ns	ns	*Rgn*	10	
1440764_at	^20430444	^20432638	ns	**1.18**	ns	**1.29**	ns	ns	*Araf*	11	
1452279_at	20502657	20508661	ns	**1.66**	**1.21**	**1.20**	ns	ns	*Cfp*	12	*a*
1447537_at	20508977	20509301	**1.15**	ns	ns	**1.50**	ns	ns	*1500032P08Rik*	13	*a*
1416242_at	22796396	22942189	**1.08**	*1.09*	ns	ns	ns	ns	*Klhl13*	14	
1448269_a_at	22796396	22942189	**1.26**	**1.94**	ns	ns	ns	ns	*Klhl13*	14	
1429028_at	33584857	33616555	**1.11**	**1.18**	ns	ns	ns	ns	*Dock11*	15	
1457265_at	34148177	34149112	**1.14**	ns	ns	*1.15*	**ns**	ns	*Sfrs17b*	16	
1417609_at	34413894	34424221	**1.25**	*1.16*	ns	ns	ns	ns	*Ube2a*	17	
1449393_at	39855741	39875274	ns	*1.05*	*1.24*	ns	ns	ns	*Sh2d1a*	18	
1438916_x_at	47908927	47909357	*1.06*	*1.12*	ns	*1.32*	ns	ns	6720401G13Rik*	19	
1435744_at	47916296	47922553	**1.19**	ns	ns	**1.34**	ns	ns	6720401G13Rik*	19	
1419033_at	48194223	48246471	**1.22**	ns	ns	*1.21*	ns	ns	*2610018G03Rik*	20	
1434678_at	48466677	48468296	**1.11**	**1.14**	ns	ns	ns	ns	*Mbnl3*^*&*^	21	
1426832_at	53752788	53761020	**1.34**	**1.13**	*1.05*	**1.41**	ns	ns	*Ddx26b*	22	
1454760_at	54307208	54320357	*1.14*	ns	ns	*1.15*	ns	ns	*Htatsf1*	23	
1455635_at	54630061	54630758	ns	*1.09*	ns	*1.23*	ns	ns	*4732460I02Rik*	24	*b*
1426863_at	54636524	54646143	**1.15**	ns	ns	*1.37*	ns	ns	*Rbmx*	25	*b*
1423369_at	65931756	65971138	**1.18**	**1.23**	ns	ns	ns	ns	*Fmr1*	26	
1451302_at	67639051	67642592	**1.18**	**1.34**	ns	ns	ns	ns	*1110012L19Rik*	27	
1435228_at	67712927	67713571	**1.18**	**1.27**	ns	ns	ns	ns	*BC023829*	28	
1418397_at	70587973	70604418	**1.25**	*1.06*	ns	ns	ns	ns	*Zfp275*	29	
1448323_a_at	70728980	70741722	**1.30**	**1.17**	ns	ns	ns	ns	Bgn*	30	
1437889_x_at	70740822	70741271	**1.17**	*1.09*	ns	ns	ns	ns	Bgn*	30	
1426677_at	71468799	71481154	**1.24**	ns	ns	*1.16*	ns	ns	*Flna*	31	
1448354_at	71654824	71674529	**1.08**	*1.09*	*1.07*	ns	ns	ns	*G6pdx*	32	
1455724_at	75694953	75696134	**1.16**	*1.07*	ns	ns	ns	ns	*Prrg1*	33	
1420514_at	78316031	78343314	**1.13**	**1.21**	ns	ns	ns	ns	*Tmem47*	34	
1417307_at	80194208	82450389	**1.20**	**1.24**	ns	ns	ns	ns	*Dmd*	35	
1448665_at	80194208	82450389	**1.20**	**1.39**	ns	ns	ns	ns	*Dmd*	35	
1423744_x_at	91434045	91435976	**1.53**	**1.37**	**1.34**	**1.38**	**1.41**	**1.37**	Eif2s3x*	36	
1451090_a_at	91434045	91435976	**1.55**	**1.45**	**1.32**	**1.43**	**1.39**	**1.39**	Eif2s3x*	36	
1421895_at	91435260	91457988	**1.48**	**1.60**	**1.34**	ns	**1.71**	**1.58**	Eif2s3x*	36	
1435818_at	91522460	91523059	**1.08**	*1.06*	ns	ns	ns	ns	*Klhl15*	37	
1434729_at	92834534	92835231	*1.06*	*1.07*	ns	ns	ns	ns	*Zc4h2*	38	
1437064_at	95514880	95516618	**1.22**	**1.56**	ns	ns	ns	ns	*Ar*	39	
1455647_at	95517026	95518558	**1.29**	**1.62**	ns	ns	ns	ns	*Ar*	39	
1419108_at	95752847	96086120	*1.06*	ns	ns	**1.10**	ns	ns	*Ophn1*	40	
1427072_at	96265193	96270067	**1.47**	**1.22**	ns	ns	ns	ns	*Stard8*	41	
1416918_at	97963090	98013749	**1.23**	*1.10*	ns	ns	ns	ns	*Dlg3*	42	
1455465_at	98394381	98395191	ns	**1.15**	ns	**1.15**	ns	ns	*--- (Snx12 intron)*	i	
1416295_a_at	98459719	98463545	**1.21**	**1.62**	ns	ns	ns	ns	*Il2rg*^*&*^	43	
1460631_at	98878217	98879690	**1.19**	*1.20*	ns	ns	ns	ns	*Ogt*	44	
1440522_at	100590759	100591430	ns	**2.02**	ns	*1.17*	ns	**ns**	*Chic1*	45	
1427262_at	100655713	100678556	**128**	**114**	**55**	**7.9**	**65**	**33**	Xist*	46	
1427263_at	100655713	100678556	**1.9**	**16.7**	**5.1**	**3.3**	**5.1**	**2.5**	Xist*	47	
1436936_s_at	100677542	100678588	**22**	**67**	**6.5**	**26**	**22**	**11**	Xist*	47	*c*
1442137_at	^100689134	^100701764	ns	ns	**1.13**	*1.07*	**1.16**	ns	2010000I03Rik (Jpx)*	48	*c*
1438838_at	100764844	100765429	*1.05*	**1.12**	ns	**1.70**	**1.40**	ns	B230206F22Rik (Ftx)*	49	*c*
1439305_at	100772965	100773674	**1.32**	**1.20**	ns	ns	**1.46**	*1.09*	*--- (Ftx intron)*	49i	*c*
1435822_at	101705781	101707391	**1.19**	**1.28**	ns	*1.16*	ns	ns	*D830012I24Rik*	50	
1438893_at	102237486	102238144	ns	ns	**1.22**	ns	**1.28**	**1.06**	5530601H04Rik*	51	
1436347_a_at	102238820	102265435	**1.06**	**1.24**	**1.41**	*1.18*	**1.63**	**1.24**	5530601H04Rik*	51	
1452750_at	^102232689	^102265463	**1.20**	**1.33**	**1.33**	**1.34**	**1.58**	**1.27**	5530601H04Rik*	51	*d*
1417921_at	102275115	102278930	**1.27**	**1.40**	**1.30**	**1.16**	**1.48**	**1.15**	2610029G23Rik*	52	*d*
1433537_at	102992735	102993644	**1.30**	ns	ns	*1.23*	ns	ns	*Atrx*	53	
1453734_at	102993154	102995719	**1.14**	*1.13*	ns	ns	ns	ns	*Atrx*	53	
1418774_a_at	103222614	103320808	**1.19**	*1.09*	ns	ns	ns	ns	*Atp7a*	54	
1436921_at	103321257	103323499	**1.21**	*1.17*	ns	ns	ns	ns	*Atp7a*	54	
1457753_at	103354989	103355829	ns	**1.11**	*1.14*	ns	ns	ns	*Tlr13*	55	
1435584_at	104368242	104369000	*1.03*	*1.07*	ns	ns	ns	ns	*A630033H20Rik*	56	
1453078_at	104978092	104980504	*1.11*	**1.26**	ns	ns	ns	ns	*2610002M06Rik*	57	
1421871_at	106290703	106357275	**1.43**	*1.22*	ns	ns	ns	ns	*Sh3bgrl*	58	
1459571_at	106306845	106307385	*1.06*	**1.15**	ns	ns	ns	ns	*--- (Sh3bgrl intron)*	58i	
1428107_at	106356680	106357810	**1.64**	*1.14*	ns	ns	ns	ns	*Sh3bgrl*	58	
1442003_at	126999308	127000367	ns	*1.18*	ns	*1.17*	ns	ns	*Diap2*	59	
1416807_at	131120240	131122656	**1.23**	ns	*1.06*	ns	ns	ns	*Rpl36a*	60	
1425914_a_at	131252558	131256449	**1.08**	*1.08*	ns	ns	ns	ns	*Armcx1*	61	
1456739_x_at	131338685	131338978	**1.64**	**1.41**	ns	ns	ns	ns	*Armcx2*	62	
1435829_at	131505913	131506643	**1.16**	**1.35**	ns	ns	ns	ns	*Zmat1*	63	
1428512_at	132424218	132425617	**1.69**	**1.21**	ns	*1.14*	ns	ns	*Bhlhb9*	64	
1428209_at	132673583	132674982	**1.84**	*1.08*	ns	ns	ns	ns	*Bex4*	65	*e*
1418171_at	132704620	132706881	**1.37**	**2.04**	ns	ns	ns	ns	*Tceal8*	66	*e*
1451230_a_at	132779633	132781675	**1.59**	*1.11*	ns	ns	ns	ns	*Wbp5*	67	
1439413_x_at	133267489	133267797	**1.14**	*1.14*	ns	ns	ns	ns	*Morf4l2*	68	
1418318_at	136145158	136207908	*1.08*	**1.22**	ns	ns	ns	ns	*Rnf128*	69	
1449036_at	136145158	136207908	*1.10*	**1.15**	ns	ns	ns	ns	*Rnf128*	69	
1456027_at	136476076	136477767	*1.06*	ns	ns	*1.10*	*1.05*	ns	*Rbm41*	70	
1416052_at	136991147	137010679	**1.11**	**1.30**	ns	ns	ns	ns	*Prps1*	71	
1425476_at	137909933	138123778	**1.19**	**1.15**	ns	ns	ns	ns	*Col4a5*	72	
1460016_at	139118850	139119428	**1.08**	*1.09*	ns	**1.22**	ns	ns	*Tmem164*	73	
1441195_at	139127416	139128111	*1.05*	ns	ns	**1.22**	ns	ns	*--- (Tmem164 intron)*	73i	
1454741_s_at	139269949	139272918	**1.25**	**1.16**	ns	ns	ns	ns	*Tmem164*	73	
1428930_at	146843122	146893683	*1.10*	ns	**1.14**	ns	**1.29**	ns	*Tmem29*	74	
1426306_a_at	147240996	147247504	ns	**1.12**	*1.08*	ns	ns	ns	*Maged2*	75	
1426497_at	148667784	148708632	ns	ns	**1.26**	ns	**1.25**	**1.13**	Kdm5c*	76	
1426498_at	148667784	148708632	ns	ns	**1.18**	ns	**1.23**	*1.07*	Kdm5c*	76	
1441449_at	148679626	148681294	ns	ns	*1.08*	*1.11*	**1.15**	ns	Kdm5c*	76	
1441450_s_at	148679626	148681294	ns	ns	**1.18**	*1.12*	**1.19**	ns	Kdm5c*	76	
1457930_at	148691371	148692025	**1.14**	**1.18**	**1.24**	ns	**1.47**	**1.21**	Kdm5c*	76	
1440123_at	148699256	148700276	**1.10**	**1.24**	**1.29**	ns	**1.51**	*1.09*	*--- (Kdm5c intron)*	76i	
1444157_a_at	148708371	148709078	*1.09*	**1.26**	**1.28**	**1.26**	**1.46**	**1.18**	Kdm5c*	76	
1444158_at	148708371	148709078	**1.28**	**1.31**	**1.24**	**1.19**	**1.44**	**1.18**	Kdm5c*	76	*f*
1435348_at	148712014	148713640	**1.24**	**1.32**	**1.33**	**1.38**	**1.53**	**1.28**	*D930009K15Rik*	77	*f*
1428499_at	148729448	148730847	*1.08*	ns	ns	*1.10*	ns	ns	*2810454L23Rik*	78	*f*
1441020_at	148742556	148743349	*1.07*	**1.14**	ns	*1.29*	ns	ns	*--- (Intergenic)*	79	*f*
1441816_at	148749758	148750124	ns	ns	**1.12**	**1.32**	ns	ns	*2900056M20Rik*	80	*f*
1433992_at	149044532	149046561	**1.15**	*1.08*	ns	ns	ns	ns	*Shroom2*	81	
1422498_at	149470708	149472081	**1.13**	**1.29**	ns	ns	ns	ns	*Mageh1*	82	
1430538_at	150158096	150175958	**1.29**	**1.36**	ns	ns	ns	ns	*2210013O21Rik*	83	
1416167_at	151758460	151773001	**1.50**	*1.17*	ns	ns	ns	ns	*Prdx4*	84	
1441360_at	155711288	155712253	ns	**1.09**	ns	**1.43**	ns	ns	*--- (Rps6ka3 intron)*	85i	
1455206_at	155803349	155806175	ns	**1.32**	ns	*1.20*	ns	ns	*Rps6ka3*	85	
1452358_at	158154967	158217429	**1.10**	**1.11**	ns	ns	ns	ns	*Rai2*	86	
1448111_at	159340115	159469264	**1.18**	**1.32**	*1.06*	ns	ns	ns	*Ctps2*	87	
1452657_at	160346948	160371598	**1.12**	**1.15**	ns	ns	ns	ns	*Ap1s2*	88	
1438953_at	160838594	160838901	**1.18**	ns	ns	*1.10*	ns	ns	*Figf*	89	
1438954_x_at	160838594	160838901	**1.20**	ns	ns	*1.11*	ns	ns	*Figf*	89	
1424124_at	161374107	161418260	*1.20*	*1.26*	ns	ns	ns	ns	*Mospd2*	90	
1423091_a_at	162676903	162826964	**1.21**	**1.12**	ns	ns	ns	ns	*Gpm6b*	91	
1415906_at	163645025	163645984	**1.35**	**1.33**	ns	ns	ns	ns	*Tmsb4x*	92	
1454843_at	163784253	163785400	**1.46**	*1.13*	ns	ns	ns	ns	*Prps2*	93	
1417704_a_at	165233904	165742756	**1.09**	*1.14*	ns	ns	ns	ns	*Arhgap6*	94	
1451867_x_at	165233904	165742367	**1.14**	**1.32**	ns	ns	ns	ns	*Arhgap6*	94	
**B. Male-biased genes**											
**ProbeSet ID**	**Start align**	**Stop align**	**Ki**	**Li**	**Lu**	**St**	**Ey**	**Hi**	**Gene symbol**		
1422827_x_at	7461369	7471588	*1.08*	ns	ns	*1.11*	ns	ns	*Slc35a2*	1	
1432533_a_at	7462961	7471586	**1.10**	*1.10*	ns	ns	ns	ns	*Slc35a2*	1	
1436664_a_at	7470132	7471590	**1.10**	**1.10**	ns	ns	ns	ns	*Slc35a2*	1	
1425490_a_at	7700426	7709678	*1.04*	**1.11**	ns	ns	ns	ns	*Wdr13*	2	
1422660_at	7719484	7722860	ns	ns	ns	ns	*1.08*	**1.18**	*Rbm3*	3	
1457519_at	7729990	7730655	**1.08**	**1.09**	ns	ns	ns	ns	*Tbc1d25*	4	
1438753_at	7779325	7780137	**1.07**	*1.10*	ns	ns	ns	ns	*--- (Porcn intron)*	i	
1451939_a_at	9616032	9694713	ns	ns	*1.11*	ns	*1.15*	ns	*Srpx*	5	
1450039_at	12648662	12749154	**1.22**	**1.18**	ns	ns	ns	ns	*Usp9x*	6	
1431955_at	20783706	20798793	**1.05**	ns	ns	*1.07*	ns	ns	*4930453H23Rik*	7	
1422241_a_at	34727595	34731158	**1.08**	*1.11*	ns	ns	ns	ns	*Ndufa1*	8	
1416344_at	35758238	35809556	**1.24**	ns	*1.03*	*1.14*	ns	ns	*Lamp2*	9	
1439298_at	39437302	39438087	**1.09**	*1.14*	ns	ns	ns	ns	*--- (Xiap intron)*	i	
1449825_at	43682183	43683528	*1.05*	**1.08**	ns	ns	ns	ns	*Actrt1*	10	
1433652_at	47135712	47137009	ns	**1.09**	ns	**1.55**	ns	ns	*Igsf1*	11	
1442549_at	48470442	48471160	ns	*1.10*	*1.19*	ns	ns	ns	*Mbnl3*^*&*^	12	
1453453_at	49321871	49325949	*1.03*	**1.08**	ns	ns	ns	ns	*1700080O16Rik*	13	
1443620_at	49403554	49404019	*1.21*	ns	*1.14*	ns	ns	ns	*Gpc4*	14	
1448736_a_at	50341268	50374837	**1.17**	**1.32**	ns	ns	ns	ns	*Hprt*	15	
1435815_at	58963393	58964128	ns	**1.16**	ns	ns	ns	**1.10**	*Ldoc1*	16	
1434739_at	66015028	66057768	**1.07**	**1.21**	ns	ns	ns	ns	*Fmr1nb*	17	
1458014_at	67115572	67116574	ns	*1.06*	ns	*1.05*	ns	ns	*Aff2*	18	
1421536_at	70070608	70083883	*1.03*	*1.05*	ns	ns	ns	ns	*Gabrq*	19	
1417412_at	70473356	70475147	**1.06**	ns	ns	*1.10*	ns	ns	*F8a*	20	
1422711_a_at	70901332	70905177	ns	**1.14**	ns	*1.19*	ns	ns	*Pnck*	21	
1451049_at	70931516	70961761	**1.13**	ns	ns	*1.13*	ns	ns	*Bcap31*	22	
1438120_x_at	71260144	71260313	**1.19**	**1.11**	ns	ns	ns	ns	*Irak1*	23	
1450161_at	71669950	71694950	**1.07**	*1.11*	ns	ns	ns	ns	*Ikbkg*	24	
1437553_at	72698339	72699357	**1.06**	*1.07*	ns	ns	ns	ns	*Brcc3*	25	
1429793_at	82514833	82515627	*1.06*	*1.09*	ns	ns	ns	ns	*1600014K23Rik*	26	
1458481_at	98456711	98459331	*1.04*	*1.10*	ns	ns	ns	ns	*Il2rg*^*&*^	27	
1447725_at	100211276	100211547	*1.06*	**1.11**	ns	ns	ns	ns	*C030034E14Rik*	28	
1437355_at	104032421	104033595	*1.04*	**1.08**	ns	ns	ns	ns	*Zcchc5*	29	
1422164_at	108009755	108012520	*1.06*	**1.17**	ns	ns	ns	ns	*Pou3f4*	30	
1444668_at	120324027	120326909	**1.13**	**1.25**	**1.15**	ns	ns	ns	*Astx*	31	
1417979_at	130385542	130400116	*1.03*	ns	ns	ns	**1.17**	ns	*Tnmd*	32	
1425954_a_at	147018680	147022624	*1.05*	**1.14**	ns	ns	ns	ns	*Apex2*	33	

The female-biased (**A**) and male-biased (**B**) genes with the same direction of sex-bias in more than one tissue are listed. The mean fold-changes for significant differences are given for each tissue. Fold-changes in Italic font denote the significance level p<0.01, and fold-changes in bold font denote the significance level p<0.001. The letters “*a-f*”, to the right in the table, indicate female-biased gene clusters as described in the main text. The additional notation used in the table is as follows. i: intronic transcript. ^: the exact probe mapping coordinates was not specified in the array annotation file, and gene start/stop locations are therefore given according to the NCBI37/mm9 genome assembly. ^&^: these genes showed opposing sex-bias for different probes. *: known XCI escapee gene.

### Candidate genes for tissue-specific escape from X-inactivation

**Figure 4:**
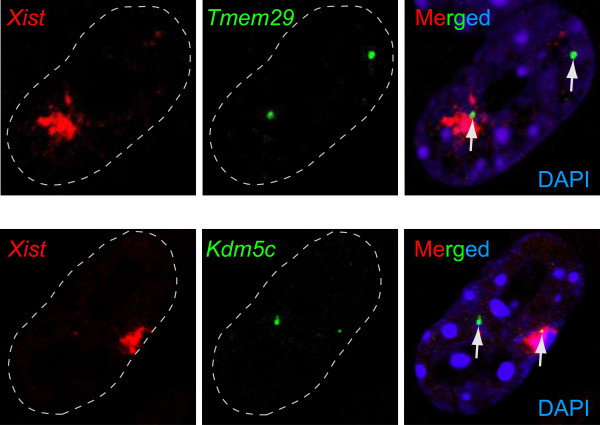
**Confirmation of a candidate XCI escapee gene.** The figure displays results from RNA-FISH experiments on female embryonic fibroblasts. The upper panel shows a cell with biallelic RNA signals for the candidate gene *Tmem29*, which escaped in 12% of the cells (82 cells counted). The lower panel shows a cell with biallelic RNA signals for a positive control for escape, *Kdm5c*, which escaped in 25% of the cells (52 cells counted).

We sought to investigate whether any of the newly identified female-biased genes might escape XCI. Candidates for this analysis were selected from Table 2, since these genes exhibit bias in at least two tissues, and therefore the evidence for possible escape from inactivation is stronger than for genes biased only in one tissue. Indeed, every gene in Table 2 that were female-biased consistently in all tissue analysed, have been previously recognised as escapees [22-25]. New selected candidates might escape XCI in a tissue-specific manner. As a first step, to identify novel escapee genes, we here investigated possible biallelic expression of five candidate genes in primary female mouse embryonic fibroblasts derived from skin. We performed RNA-FISH using a probe for *Xist* RNA in parallel with a probe for either one of the five candidate genes (*4732460I02Rik*/*Rbmx*^*24*/*25*^, *Rbm4*^70*^, *Tmem164*^*73*^, *Tmem29*^*74*^ and *Ctps2*^*87*^; superscripted numbers corresponding to Table 2) and a positive control for escape: *Kdm5c*^*76*^. *Kdm5c* showed biallelic signals in 25% of the cells (13 of 52 counted, Figure 4). One of the candidates, *Tmem29*, was confirmed to partially escape XCI, showing biallelic signals in 12% of the cells (10 of 82 counted, Figure 4). None of the other candidates indicated biallelic expression in any of the examined cells, suggesting that they are X-inactivated in mouse embryonic skin fibroblasts. The possibility remains that they might escape inactivation in other cell types.

### Gene ontology analysis

To investigate whether sex-biased X-linked genes are associated with a specific functionality that is overrepresented, we performed a Gene Ontology classification [26,27]. To do this, we contrasted the proportion of sex-biased genes in each functional category with the proportion of the same functional category among all genes in the genome (Additional file 3 A) or with all genes on the X-chromosome (Additional file 3 B). These analyses revealed three groups with significant over-representation (p<0.05 after Bonferroni correction) in the genome-wide comparison, namely **IPR006911**: **Protein of unknown function DUF634** (p=3E-06; overrepresented among female-biased genes in kidney), **GO**:**0031090 Organelle membrane** (p=0.009; overrepresented among male-biased genes in kidney), and perhaps most interesting because of possible sex differences in synaptic plasticity: **mmu04720**: **Longterm potentiation** (p=0.04; among female-biased genes in striatum).

### Female-biased X-genes pre-date mammalian radiation

**Figure 5:**
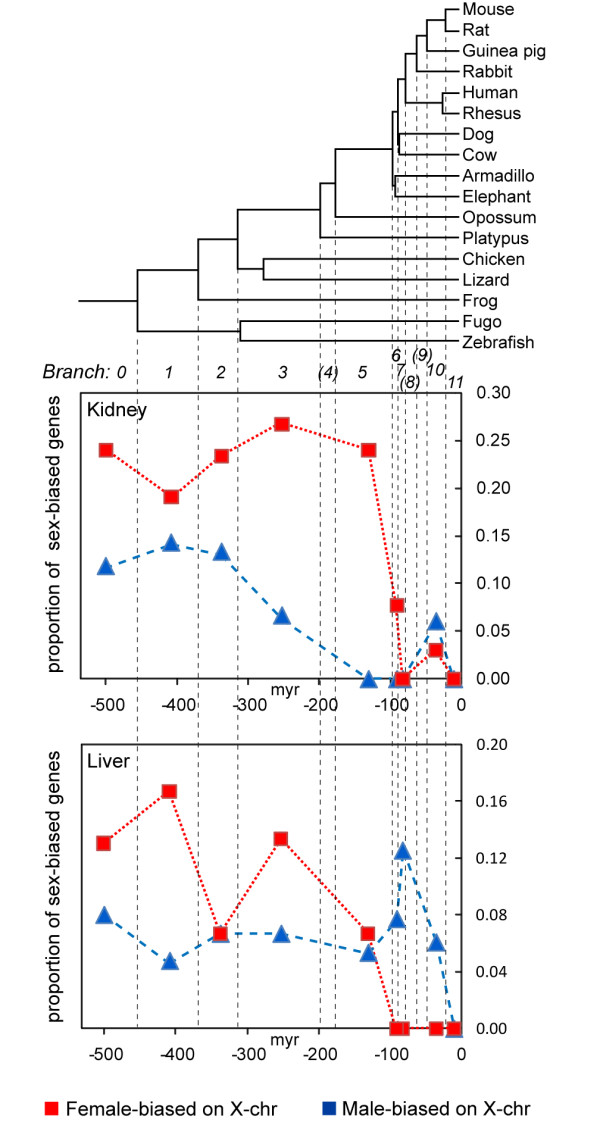
**Age of sexually dimorphic genes on the X.** The upper panel shows a phylogeny and gene branch assignment according to Zhang et al. [28]. The lower panels show the proportion of genes with female-bias (red) and male-bias (blue) among genes assigned to each branch, for the kidney and liver datasets. Branches marked with parenthesis (4, 8 and 9) are not included due to low number of probes corresponding to these branches (see Methods).

It was earlier shown that genes on the X-chromosome with female-biased function (defined by high expression in female reproductive tissues) are mainly evolutionarily old genes, while genes with male-biased function (defined by high expression in male reproductive tissues) are enriched among more recently formed genes (<50 myr) [28]. To investigate whether similar conclusions hold for X-genes with sex-biased expression in non-reproductive tissues, we determined the age of the X-linked genes included in our dataset. To do this, we used gene age data obtained recently by comparative genomic analysis for the presence or absence of orthologs in a vertebrate phylogeny [28], and we calculated the proportion of female- and male-biased genes among the genes assigned to each branch in the phylogeny. We found that the proportion of genes with female-biased expression in kidney and liver was higher among evolutionarily old genes, preceding the radiation of placental mammals, than among younger genes (Figure 5). The difference in proportion of female-biased genes older than 100 myr (branch 0–5) versus younger genes (<100 myr, branch 6–11) was significant both in kidney (p=1.6E-6, two-sided Fischer’s exact test) and liver (p=1.9E-4). A modestly significant age-related decrease in male-biased genes was observed in kidney (p=0.032), while no significant difference was found in liver (p=0.64). Similar analyses of the other somatic tissues included in our study were not possible due to the low number of sex-biased genes detected in those tissues.

## Discussion

### Skewed allocation of male- and female-biased X-genes in somatic tissues

We have used a large sample set of oligonucleotide microarrays to identify with high-resolution transcripts encoded on the X-chromosome that are expressed in a sex-biased manner in somatic tissues. We observed a skewed allocation of sex-biased somatic transcripts encoded on the X-chromosome: namely an abundance of female-biased transcripts and a paucity of male-biased transcripts. A previous large genome-wide analysis in mice, including four somatic tissues (liver, adipose, muscle and brain) also found over-representation of female-biased genes on the X [12]. However, under-representation of male-biased genes was not reported, possibly due to the lower probe content of their arrays. Our study might thus provide the first empirical evidence of de-masculinisation of a mammalian X-chromosome in terms of X-gene expression in somatic tissues. Our investigation of somatic tissues complements earlier analyses of sex-biased gene expression on the X-chromosome that investigated expression in reproduction-related tissues such as testis, ovary and placenta [29]. Khil et al. demonstrated that genes enriched in mouse testis are under-represented on the X-chromosome, likely as a consequence of meiotic sex chromosome inactivation (MSCI), and at the same time, genes with high expression in ovary and placenta were shown to be enriched [29]. We here demonstrated a similarly skewed allocation of male- and female-biased X-genes in somatic tissues. However, a crucial difference is that the observed paucity of male-biased genes in somatic tissues is unlikely to be explained by MSCI, since this process does not occur in these tissues. The results are relevant in terms of interpreting the Rice hypothesis [9]. This hypothesis states that any recessive allele on the X that gives males a reproductive advantage is immediately available for positive selection since males carry a single X-chromosome. Accordingly, an excess of male-beneficial genes should accumulate on the X. This may be the case for genes specifically expressed in spermatogonia [30], but seemingly not for genes enriched in whole testis [29] or for genes expressed in somatic tissues, as indicated in our current study. Therefore, it is most plausible that different selection pressures operate on X-genes active in different tissues, and that the final result in terms of the evolution of gene composition in the X (masculinisation versus feminisation) is a compromise between different evolutionary forces acting on reproductive and somatic tissues, as has been hypothesised both for rodents [8] and similarly for *Drosophila*[31]. Our data gives support to this hypothesis.

The importance of our results can also be discussed in relationship to studies of gene composition of the sex chromosomes of other species. Indeed, the paucity of male-biased genes in both reproductive and somatic tissues of the mouse is similar to that reported in *Drosophila melanogaster*[10] and *Caenohabditis elegans*[11]. Interestingly, while the overall pattern is the same in many species, the particular genes are not. Moreover, the collections of genes biased in reproductive tissues are different from those found in somatic tissues from the same species [32,33]. Nonetheless, the similarity in our results from the mouse X-chromosome and in the evolutionarily unrelated X-chromosomes in distant species such as *Drosophila* and *Caenohabditis elegans*, indicate the generality of at least some selective forces acting on X-chromosomes.

### Genes female-biased in multiple tissues reveal genes that escape X-inactivation

The analysis of genes with sex-bias in more than one tissue revealed clues to the potential mechanisms controlling sex-bias of X-encoded genes as well as to whether such mechanisms operate in a ubiquitous versus tissue-specific fashion. Interestingly, while several of the genes listed in Table 2were female-biased in all six tissues, none of the male-biased X-linked transcripts were consistently biased in four or more tissues. Indeed, only two transcripts, *Lamp2* and *Astx*, were male-biased in more than two tissues. This was in stark contrast to the widespread expression differences of several female-biased transcripts, with 25 unique genes female-biased in more than two tissues (Table 2). These observations are in agreement with the conclusion that the mechanisms controlling female-bias versus male-bias are different. It is worth noting that all transcripts that were female-biased in all tissues were encoded by genes known to escape XCI [22-25], namely *Ddx3x*, *Kdm6a*, *Eif2s3x*, *Xist*, *5530601H04Rik*, *2610029G23Rik*, *Kdm5c*, and *D930009K15Rik*. Our microarray analysis of somatic tissues, together with previous global analysis of escape from XCI performed on fibroblasts *in vitro*[23], indicate that most of the ubiquitously expressed coding escapee genes in mouse have now been identified. On the other hand, the fact that most of the genes included in Table 2were female-biased in a tissue-specific manner, might suggest that escape from XCI is conveyed by tissue-specific mechanisms, at least for some genes.

Our RNA-FISH experiments confirmed that *Tmem29* escapes XCI, adding yet another gene to the 15 or so escapees previously known in mouse [34]. *Tmem29* escaped inactivation in 12% of the fibroblasts counted, while *Kdm5c* presented biallelic expression in 25% of the cells. This deviation suggests dissimilarity in control mechanisms for biallelism for these two genes, but we cannot fully rule out that differences in probe composition contributed to the divergence observed.

Genes escaping X-inactivation [35] and even the Xi heterochromatin itself [36] may underlie significant phenotypic differences between the sexes. It should however be pointed out that female mice that are monosomic for X are fertile and show a mild phenotype compared to women with Turner Syndrome [37].

### Small female-biased gene clusters on X

**Figure 6:**
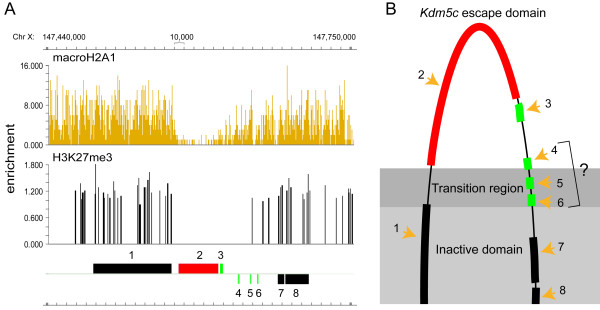
**A Model of the *Kdm5c *escape domain.A:** Depletion of the repressive histone marks macroH2A1 and H3K27me3 in the *Kdm5c* domain (liver ChIP data: GSM469459 and GSM517918). Genes: 1: *Iqseq2*, 2: *Kdm5c*, 3: *D930009K15Rik*, 4: *2810454L23Rik*, 5: *1441020*_*at* (*Intergenic*), 6: *2900056M20Rik*, 7: *Tspyl2*, 8: *Gpr173*. **B:** The schematic figure places *Kdm5c* (red) and the nearby-located female-biased non-coding genes (green) in a loop-out escape model, based on a general model of escape domains [40]. The light grey area symbolises a transcriptionally silenced X-inactive territory. The chromosomal region containing the known escapee genes *Kdm5c* (2) and *D930009K15Rik* (3) is depleted of repressive marks and loops out from the silenced territory. *2810454L23Rik* (4), *1441020*_*at* (5) and *2900056M20Rik* (6) are situated in the transition region between active and inactive chromosomal domains, producing variable and less robust female-bias. The coding genes located upstream (*Iqsec2*) and downstream (*Tspyl2*) of the *Kdm5c* escape domain have previously been shown to be inactivated (black) [39].

Our current investigation extended our previous discovery of female-biased gene clusters on the mouse X-chromosome [22]. Here we investigate in more detail the cluster including *Kdm5c*, because it contains the largest number of female-biased non-coding genes. X-inactive repressive histone marks, such as histone variant macroH2A1 (macroH2A1) and tri-methylation at lysine 27 of histone 3 (H3K27me3), are known to be depleted in escape domains [23,24]. It was previously shown by us and others that *Kdm5c* and *D930009K15Rik* are located in a chromosomal region virtually depleted of these repressive marks, and that both escape XCI [22-25]. Novel in the current study is the identification of three additional female-biased non-coding genes in this cluster, located downstream of *Kdm5c* and *D930009K15Rik*, apparently in the transition region of macroH2A1 and H3K27me3 enrichment (Figure 6A). This pattern is consistent with a model for the *Kdm5c* escape domain, in which *Kdm5c* and *D930009K15Rik* are located in a transcriptionally active chromosomal domain while the subsequent non-coding genes are located in the transition region between active and silenced domains (Figure 6B). We note that one of these non-coding genes, *2900056M20Rik*, was in a previous study classified as X inactivated [38], perhaps indicating tissue variability in escape among genes in the transition region. The coding genes located upstream (*Iqsec2*) and downstream (*Tspyl2*) of the female-biased *Kdm5c* cluster have previously been shown to be X-inactivated [39], and therefore the limits of the *Kdm5c* escape domain are established. Whether the non-coding RNAs encoded in this escaping domain serve any function in the regulation of escape, the preservation of the boundary between active/inactive domains, or the silencing of neighbouring inactive genes, remains to be explored.

### Other mechanisms that may explain sex-biased expression of X-encoded genes

Escape from XCI is not the only mechanism that leads to female-biased expression of X-linked genes. For example, some of the female-biased genes may be controlled by regulators acting in trans, such as hormonally regulated factors. If this is so, the abundance of female-biased genes on X may correlate with an overrepresentation of the corresponding transcription factor binding sites in X-gene promoters. Conversely, it is conceivable that X-gene transcriptional down-regulating factors are enriched in males, and binding motifs for these factors are then similarly expected to be enriched on the X. Sex-bias of some of the identified genes may also reflect sex differences in cell composition within the tissues. Another possible mechanism for sex-biased gene expression is genomic imprinting of X-chromosome genes [41,42]. Indeed, widespread sex-specific parent-of-origin-dependent allelic expression was reported recently in the brain of rodents in several studies [43-46]. This may also mediate or resolve sexually antagonistic effects [47]. If any of the mechanisms described above would affect one sex but not the other, the dosage compensation achieved by random X-inactivation would be altered, resulting in sex-biased expression in the particular tissue in which the mechanism operates.

### Female-biased X-genes are evolutionarily old

Multiple studies have demonstrated that different evolutionary processes and patterns are observed on X-chromosome and autosomal chromosomes [48]. For example, a recent study showed that two bursts of gene gain on the X-chromosome occurred during the evolution of mammals [28]. Moreover, it was suggested that the X-chromosome recently acquired a burst of young male-biased genes (operating in testis), which is consistent with a fixation of recessive male-beneficial alleles by sexual antagonism [28]. Our results provide a different story in terms of the evolution of sex-biased genes acting in somatic tissues. While both studies indicate that female-biased genes are old, the results for male-biased genes differ between our study of somatic tissues and the study of gonadal expression. We found a moderately significant abundance of evolutionarily old genes also among male-biased genes, at least in the kidney. These combined results demonstrate that considerations on the potential effect of sexual antagonism during the evolution of X-chromosome will require a concerted analysis of the selective forces operating both in gonads and in somatic tissues.

## Conclusions

We performed a high-resolution study of sex-biased RNA expression of genes located on the mouse X-chromosome in somatic tissues. We found that the mouse X-chromosome is significantly depleted of male-biased genes and enriched with female-biased genes. Our results in mice correlate with previous analyses of evolutionarily unrelated X-chromosomes in distant species such as *Drosophila melanogaster* and *Caenohabditis elegans*, indicating the generality of at least some of the selective forces acting on X-chromosomes. The expression of previously known genes that escape X-chromosome inactivation could explain only a minor part of the observed overrepresentation of female-biased genes in our data set, indicating either the existence of many tissue-specific escapees or the existence of additional sex-skewed gene regulatory mechanisms operating on the X-chromosome. Among the newly identified female-biased genes, using RNA-FISH, the gene *Tmem29* was shown to escape X-inactivation in a small proportion of female fibroblasts interrogated. An analysis of gene age showed that evolutionarily old genes, pre-dating the radiation of placental mammals, are more frequently female-biased than younger genes. A similar pattern was observed among male-biased genes in kidney, contrasting with previously shown recent gain on the X-chromosome of male-biased genes expressed in testis.

## Methods

### Data analysis

We used array data (Affymetrix Mouse 430v.2) from GeneNetwork (University of Tennessee), available at: http://www.genenetwork.org. *Datasets*: *Kidney*: “Mouse kidney M340v2 Male (Aug06) RMA” (GN240), “Mouse kidney M340v2 Female (Aug06) RMA” (GN239); *Liver*: “GenEx BXD Sal Liver Affy M430 2.0 (Feb11) RMA Males” (GN311), "GenEx BXD Sal Liver Affy M430 2.0 (Feb11) RMA Females" (GN312); *Lung*: “HZI LungM430v2 (Apr08) RMA” (GN160); *Striatum*: “HBP Rosen Striatum M340V2 (Apr05) RMA” (GN69); *Eye*: “Hamilton Eye Institute Mouse Eye M430v2 Data Set (Sept08) RMA” (GN207); *Hippocampus*: “Hippocampus Consortium M340v2 (Jun06) RMA” (GN110). The number of biological specimens was 2–6 individuals per array, as specified for each dataset in the GeneNetwork database. The arrays were normalised with Robust Multichip Average (RMA), and intensities were log-transformed and standardised to a mean of 8 and a standard deviation of 2.

Differentially expressed (DE) genes were identified in each tissue using the Wilcoxon Mann-Whitney test (R v2.6.2 Environment for Statistical Computing http://www.r-project.org, wilcox.test(); standard approximation of p-values) at p<0.001 or p<0.01 (not adjusted for multiple testing), as described in the results section. Non-parametric (Wilcoxon) and parametric (t-test) tests showed similar results in number of DE probes. Significant DE X-linked probes using Wilcoxon (or t-test) were as follows for each tissue. Kidney: 287 (290), Liver: 176 (142), Lung: 48 (45), Striatum: 38 (28), Eye: 38 (35), Hippocampus: 25 (25). The Wilcoxon test was chosen to avoid violations of the assumption of normal distribution of probe intensities in each gene. The results for all probes (using both statistics) are available in Additional file 1 and Additional file 2. Since body weight and hormonal state were not controlled, the regulatory origin of any differential expression may be the compound effects of chromosomal constitution, hormonal differences and metabolic states between the sexes. The selection criterion for probes in Table 2was a consistent (defined as a significant deviation in the same direction) female-bias or male-bias (p<0.01) in more than one tissue. SignalMap v1.9.0.03 (NimbleGen) was used to visualise the distribution of sex-biased genes shown in Figure 2. The statistical significance of the observed over-/under-representation of chromosomal location for sex-biased genes was determined with a two-sided Fisher’s exact test and the significance criterion p<0.01. For the analysis shown in Figure 3B (X’), we excluded the following known escapees: *Xist*, *Shroom4*, *Mid1*, *Kdm6a*, *Kdm5c*, *Jpx*, *Ftx*, *Eif2s3x*, *Ddx3x*, *Car5b*, *Bgn*, *BC022960*, *D930009K15Rik*, *6720401G13Rik*, *5530601H04Rik*, *2610029G23Rik*, and *1810030O07Rik*[22-25,49,50]. The female mouse liver H3K27me3 [23] and macroH2A1 [24] data used in Figure 6 were downloaded from the Gene Expression Omnibus ( http://www.ncbi.nlm.nih.gov/geo, Accession: GSE20617: GSM517918, GSE18963: GSM469459), and SignalMap v1.9.0.03 (NimbleGen) was used to visualize enrichment. For the gene age analysis, branch assignment of sex-biased genes was set according to Zhang et al. (Additional file 2) [28], matched by Ensembl ID, and the phylogeny in the upper section of Figure 5 was depicted accordingly (Zhang et al. 2010, page 2). The lower part of Figure 5 shows only those branches that were represented by at least 10 probes on the Mouse 430v.2 array. An apparent decrease in the proportion of sex-biased genes was observed around the split between branch 5 and 6. A two-sided Fisher’s exact test was applied to determine the statistical significance of the differences in the proportion of sex-biased genes assigned to branches before or after this split. GO analysis was performed in DAVID Bioinformatics Resources 6.7, NIAID/NIH [26,27], using two reference sets: all X-chromosome genes in the Mouse 430v.2 array, or all genes included in the array, independent of chromosomal location, as specified in Additional file 3 A-E.

### RNA-FISH

Mouse embryonic fibroblasts (MEFs) derived from skin were cultured in Minimum Essential Medium Eagle (Sigma) supplemented with 2% L-glutamine, 10% fetal bovine serum and 1.5% penicillin/streptomycin (Invitrogen). Upon reaching confluence, MEFs were subcultured 1:4 in Culturewell MultiWell cell culture system (Molecular Probes) and grown until reaching 80% confluence. Cultures were maintained at 37°C and 5% CO_2_. Cells were fixed by 3% paraformaldehyde for 15 min at room temperature, followed by permeabilization in 0.5% TritonX-100 in PBS with 10 mM Ribonucleoside Vanadyl Complex (New England Biolabs) for 5 min. Fixed cells were stored in 70% ethanol at −20°C until usage. Probes (*Xist*: RP23-84A16, *Tmem29*: RP23-84G23, *Tmem164*: RP23-239E14, *Kdm5c*: RP23-202H16, *Ctps2*: RP23-106K4, *Rbm41*: RP23-381C12, *4732460I02Rik*/*Rbmx*: RP23-44G23) were labeled with ChromaTide Alexa Fluor 488-5-dUTP (Molecular Probes) or Cy3-dCTP (Amersham) PA53021, using the BioPrime Array CGH Genomic Labeling system (Invitrogen). Hybridisation with labelled DNA (40 ng/μl) and mouse Cot1 DNA (100 ng/μl) (Invitrogen) was performed overnight at 38°C in 2×SSC, 50% formamide and 12% dextran sulfate, with 10 mM Ribonucleoside Vanadyl Complex. Cells were washed with 2×SSC and 50% formamide (3x5 min; 40°C) and 2×SSC (3x5 min; 40°C). Slides were mounted with Vectashield (Vector Labs). Cell imaging was carried out using a confocal microscope (Zeiss LSM 510 META, Göttingen, Germany). The images presented in Figure 4 were uniformly processed and merged in Adobe Photoshop CS3 (Adobe).

## Competing interests

The authors declare that they have no competing interests.

## Authors’ contributions

Conceived the study: BR, EJ. Prepared the microarray data: RWW. Analysed the data: BR. Participated in the RNA-FISH experiments: BR, MMJ, KJR, GKP, CK. Wrote the paper: BR, EJ, EHM, RS. All authors read and approved the final manuscript.

## Public data

Gene Expression Omnibus ( http://www.ncbi.nlm.nih.gov/geo, Accession: GSM469459, GSM517918).

GeneNetwork ( http://www.genenetwork.org, Accession: GN69, GN110, GN160, GN207, GN239, GN240, GN311, GN312).

## Supplementary Material

Additional file 1 **Microarray results: genes on the X-chromosome.** The table shows the statistics from the sex-specific analysis for probes mapping to the X-chromosome. “Biotype” is given according to Ensembl (BioMart:Ensembl Genes 62/NCBIM37), “Branch” according to Zhang et al. [28]. The colour code indicates the following significance: p<0.01; red: female-biased, blue: male-biased. This level of significance was marked to facilitate interpretation of the table, but note that the significance criterion used in the main study was p<0.001. Click here for file

Additional file 2 **Microarray results: all chromosomal locations.** The table shows the statistics from the sex-specific analysis for all probes present on the Affymetrix mouse 430v.2 array. The colour code indicates the following significance: p<0.01; red: female-biased, blue: male-biased. This level of significance was marked to facilitate interpretation of the table, but note that the significance criterion used in the main study was p<0.001. Click here for file

Additional file 3 **A-E. Gene Ontology analysis.** Results from a Gene Ontology analysis of female- and male-biased X-linked and autosomal genes, using DAVID Bioinformatics Resources 6.7, NIAID/NIH [26,27]. The GO analysis was performed using as reference either X-genes or all genes included in the microarray, as specified in the title of each table. Click here for file

## References

[B1] Mank JE Sex chromosomes and the evolution of sexual dimorphism: lessons from the genome Am Nat 2009 173 2 141 150 10.1086/595754 20374139

[B2] Lande R Sexual Dimorphism, Seexual Selection, and Adaptation in Polygenic Characters Evolution 1980 34 2 292 305 10.2307/2407393 28563426

[B3] Albert AY Otto SP Sexual selection can resolve sex-linked sexual antagonism Science 2005 310 5745 119 121 10.1126/science.1115328 16210543

[B4] Gurbich TA Bachtrog D Gene content evolution on the X chromosome Curr Opin Genet Dev 2008 18 6 493 498 10.1016/j.gde.2008.09.006 18929654 PMC4590997

[B5] Innocenti P Morrow EH The sexually antagonistic genes of Drosophila melanogaster PLoS Biol 2010 8 3 e1000335 10.1371/journal.pbio.1000335 20305719 PMC2838750

[B6] Connallon T Clark AG The resolution of sexual antagonism by gene duplication Genetics 2011 187 3 919 937 10.1534/genetics.110.123729 21220356 PMC3063682

[B7] Bachtrog D A dynamic view of sex chromosome evolution Curr Opin Genet Dev 2006 16 6 578 585 10.1016/j.gde.2006.10.007 17055249

[B8] Reinke V Sex and the genome Nat Genet 2004 36 6 548 549 10.1038/ng0604-548 15167926

[B9] Rice WR Sex chromosomes and the evolution of sexual dimorphism Evolution 1984 38 735 742 10.2307/2408385 28555827

[B10] Parisi M Nuttall R Naiman D Bouffard G Malley J Andrews J Eastman S Oliver B Paucity of genes on the Drosophila X chromosome showing male-biased expression Science 2003 299 5607 697 700 10.1126/science.1079190 12511656 PMC1363366

[B11] Reinke V Smith HE Nance J Wang J Van Doren C Begley R Jones SJ Davis EB Scherer S Ward S A global profile of germline gene expression in C. elegans Mol Cell 2000 6 3 605 616 10.1016/S1097-2765(00)00059-9 11030340

[B12] Yang X Schadt EE Wang S Wang H Arnold AP Ingram-Drake L Drake TA Lusis AJ Tissue-specific expression and regulation of sexually dimorphic genes in mice Genome Res 2006 16 8 995 1004 10.1101/gr.5217506 16825664 PMC1524872

[B13] Lin H Gupta V Vermilyea MD Falciani F Lee JT O'Neill LP Turner BM Dosage compensation in the mouse balances up-regulation and silencing of X-linked genes PLoS Biol 2007 5 12 e326 10.1371/journal.pbio.0050326 18076287 PMC2121114

[B14] Nguyen DK Disteche CM Dosage compensation of the active X chromosome in mammals Nat Genet 2006 38 1 47 53 10.1038/ng1705 16341221

[B15] Lyon MF Gene action in the X-chromosome of the mouse (Mus musculus L.) Nature 1961 190 372 373 10.1038/190372a0 13764598

[B16] Fry JD The genomic location of sexually antagonistic variation: some cautionary comments Evolution 2010 64 5 1510 1516 19922443 10.1111/j.1558-5646.2009.00898.x PMC3654548

[B17] Shapiro LJ Mohandas T Weiss R Romeo G Non-inactivation of an x-chromosome locus in man Science 1979 204 4398 1224 1226 10.1126/science.156396 156396

[B18] Migeon BR Some insights into X chromosome inactivation from studies of human cells Ann Endocrinol (Paris) 1980 41 4 275 280 7212635

[B19] Goldman MA Stokes KR Idzerda RL McKnight GS Hammer RE Brinster RL Gartler SM A chicken transferrin gene in transgenic mice escapes X-chromosome inactivation Science 1987 236 4801 593 595 10.1126/science.2437652 2437652

[B20] Brown CJ Willard HF Localization of a gene that escapes inactivation to the X chromosome proximal short arm: implications for X inactivation Am J Hum Genet 1990 46 2 273 279 2301397 PMC1684990

[B21] Mank JE The W, X, Y and Z of sex-chromosome dosage compensation Trends Genet 2009 25 5 226 233 10.1016/j.tig.2009.03.005 19359064 PMC2923031

[B22] Reinius B Shi C Hengshuo L Sandhu KS Radomska KJ Rosen GD Lu L Kullander K Williams RW Jazin E Female-biased expression of long non-coding RNAs in domains that escape X-inactivation in mouse BMC Genomics 2010 11 614 10.1186/1471-2164-11-614 21047393 PMC3091755

[B23] Yang F Babak T Shendure J Disteche CM Global survey of escape from X inactivation by RNA-sequencing in mouse Genome Res 2010 20 5 614 622 10.1101/gr.103200.109 20363980 PMC2860163

[B24] Changolkar LN Singh G Cui K Berletch JB Zhao K Disteche CM Pehrson JR Genome-wide distribution of macroH2A1 histone variants in mouse liver chromatin Mol Cell Biol 2010 30 23 5473 5483 10.1128/MCB.00518-10 20937776 PMC2976432

[B25] Lopes AM Arnold-Croop SE Amorim A Carrel L Clustered transcripts that escape X inactivation at mouse XqD Mamm Genome 2011 10.1007/s00335-011-9350-6 21769671

[B26] da Huang W Sherman BT Lempicki RA Systematic and integrative analysis of large gene lists using DAVID bioinformatics resources Nat Protoc 2009 4 1 44 57 19131956 10.1038/nprot.2008.211

[B27] da Huang W Sherman BT Lempicki RA Bioinformatics enrichment tools: paths toward the comprehensive functional analysis of large gene lists Nucleic Acids Res 2009 37 1 1 13 10.1093/nar/gkn923 19033363 PMC2615629

[B28] Zhang YE Vibranovski MD Landback P Marais GA Long M Chromosomal redistribution of male-biased genes in mammalian evolution with two bursts of gene gain on the X chromosome PLoS Biol 2010 8 10 e1000494 10.1371/journal.pbio.1000494 20957185 PMC2950125

[B29] Khil PP Smirnova NA Romanienko PJ Camerini-Otero RD The mouse X chromosome is enriched for sex-biased genes not subject to selection by meiotic sex chromosome inactivation Nat Genet 2004 36 6 642 646 10.1038/ng1368 15156144

[B30] Wang PJ McCarrey JR Yang F Page DC An abundance of X-linked genes expressed in spermatogonia Nat Genet 2001 27 4 422 426 10.1038/86927 11279525

[B31] Parsch J X chromosome: expression and escape PLoS Genet 2009 5 11 e1000724 10.1371/journal.pgen.1000724 19936021 PMC2770632

[B32] Jiang M Ryu J Kiraly M Duke K Reinke V Kim SK Genome-wide analysis of developmental and sex-regulated gene expression profiles in Caenorhabditis elegans Proc Natl Acad Sci USA 2001 98 1 218 223 10.1073/pnas.98.1.218 11134517 PMC14571

[B33] Thoemke K Yi W Ross JM Kim S Reinke V Zarkower D Genome-wide analysis of sex-enriched gene expression during C. elegans larval development Dev Biol 2005 284 2 500 508 10.1016/j.ydbio.2005.05.017 15987632

[B34] Yang C Chapman AG Kelsey AD Minks J Cotton AM Brown CJ X-chromosome inactivation: molecular mechanisms from the human perspective Hum Genet 2011 130 175 85 10.1007/s00439-011-0994-9 21553122

[B35] Disteche CM Escape from X inactivation in human and mouse Trends Genet 1995 11 1 17 22 10.1016/S0168-9525(00)88981-7 7900190

[B36] Wijchers PJ Yandim C Panousopoulou E Ahmad M Harker N Saveliev A Burgoyne PS Festenstein R Sexual dimorphism in mammalian autosomal gene regulation is determined not only by Sry but by sex chromosome complement as well Dev Cell 2010 19 3 477 484 10.1016/j.devcel.2010.08.005 20833369

[B37] Burgoyne PS Baker TG Oocyte depletion in XO mice and their XX sibs from 12 to 200 days post partum J Reprod Fertil 1981 61 1 207 212 10.1530/jrf.0.0610207 7452619

[B38] Tsuchiya KD Greally JM Yi Y Noel KP Truong JP Disteche CM Comparative sequence and x-inactivation analyses of a domain of escape in human xp11.2 and the conserved segment in mouse Genome Res 2004 14 7 1275 1284 10.1101/gr.2575904 15197169 PMC442142

[B39] Li N Carrel L Escape from X chromosome inactivation is an intrinsic property of the Jarid1c locus Proc Natl Acad Sci USA 2008 105 44 17055 17060 10.1073/pnas.0807765105 18971342 PMC2579377

[B40] Berletch JB Yang F Xu J Carrel L Disteche CM Genes that escape from X inactivation Hum Genet 2011 130 2 237 245 10.1007/s00439-011-1011-z 21614513 PMC3136209

[B41] Skuse DH James RS Bishop DV Coppin B Dalton P Aamodt-Leeper G Bacarese-Hamilton M Creswell C McGurk R Jacobs PA Evidence from Turner's syndrome of an imprinted X-linked locus affecting cognitive function Nature 1997 387 6634 705 708 10.1038/42706 9192895

[B42] Skuse DH Genomic imprinting of the X chromosome: a novel mechanism for the evolution of sexual dimorphism J Lab Clin Med 1999 133 1 23 32 10.1053/lc.1999.v133.a94575 10385478

[B43] Wang X Sun Q McGrath SD Mardis ER Soloway PD Clark AG Transcriptome-wide identification of novel imprinted genes in neonatal mouse brain PLoS One 2008 3 12 e3839 10.1371/journal.pone.0003839 19052635 PMC2585789

[B44] Gregg C Zhang J Weissbourd B Luo S Schroth GP Haig D Dulac C High-resolution analysis of parent-of-origin allelic expression in the mouse brain Science 2010 329 5992 643 648 10.1126/science.1190830 20616232 PMC3005244

[B45] Wang X Soloway PD Clark AG Paternally biased X inactivation in mouse neonatal brain Genome Biol 2010 11 7 R79 10.1186/gb-2010-11-7-r79 20663224 PMC2926790

[B46] Gregg C Zhang J Butler JE Haig D Dulac C Sex-specific parent-of-origin allelic expression in the mouse brain Science 2010 329 5992 682 685 10.1126/science.1190831 20616234 PMC2997643

[B47] Bonduriansky R Chenoweth SF Intralocus sexual conflict Trends Ecol Evol 2009 24 5 280 288 10.1016/j.tree.2008.12.005 19307043

[B48] Vicoso B Charlesworth B Evolution on the X chromosome: unusual patterns and processes Nat Rev Genet 2006 7 8 645 653 10.1038/nrg1914 16847464

[B49] Tian D Sun S Lee JT The long noncoding RNA, Jpx, is a molecular switch for X chromosome inactivation Cell 2010 143 3 390 403 10.1016/j.cell.2010.09.049 21029862 PMC2994261

[B50] Chureau C Chantalat S Romito A Galvani A Duret L Avner P Rougeulle C Ftx is a non-coding RNA which affects Xist expression and chromatin structure within the X-inactivation center region Hum Mol Genet 2011 20 4 705 718 10.1093/hmg/ddq516 21118898

