# Supplementary material for: Abundance of female-biased and paucity of male-biased somatically expressed genes on the mouse X-chromosome
Source: BMC Genomics. 2012 Nov 10;13:607. doi: 10.1186/1471-2164-13-607 (PMC3534601; doi:10.1186/1471-2164-13-607)
Supplement: Additional file 2 — Microarray results: all chromosomal locations. The table shows the statistics from the sex-specific analysis for all probes present on the Affymetrix mouse 430v.2 array. The colour code indicates the following significance: p<0.01; red: female-biased, blue: male-biased. This level of significance was marked to facilitate interpretation of the table, but note that the significance criterion used in the main study was p<0.001. [file 1471-2164-13-607-S2.doc]

**Additional file 2**

*Editor’s note.* This file is available on *Genome Biology*’s ftp server at <ftp://ftp.biomedcentral.com/Reinius_MsID9045696646456501/>.

Username: GNBiology

Password:RedGene24
